# Orexin-A Exerts Neuroprotective Effects via OX1R in Parkinson’s Disease

**DOI:** 10.3389/fnins.2018.00835

**Published:** 2018-11-15

**Authors:** Mei-Fang Liu, Yan Xue, Cui Liu, Yun-Hai Liu, Hui-Ling Diao, Ying Wang, Yi-Peng Pan, Lei Chen

**Affiliations:** ^1^Department of Physiology, Shandong Provincial Key Laboratory of Pathogenesis and Prevention of Neurological Disorders, Qingdao University, Qingdao, China; ^2^College of Pharmacy, Jining Medical University, Rizhao, China

**Keywords:** Parkinson’s disease, orexin-A, MPTP parkinsonian model, behavior, BDNF

## Abstract

Parkinson’s disease (PD) is a common neurodegenerative disorder characterized by progressive and selective death of dopaminergic neurons. Orexin-A is involved in many biological effects of the body. It has been reported that orexin-A has protective effects in cellular models of PD. However, little is known about the protective effects of orexin-A in animal parkinsonian models and the cellular mechanism has not yet been fully clarified. The aim of this study was to evaluate the effects of orexin-A in MPTP mice model of PD as well as the possible neuroprotective mechanisms of orexin-A on dopaminergic neurons. The results from animal experiments demonstrated that orexin-A attenuated the loss of dopaminergic neurons and the decrease of tyrosine hydroxylase (TH) expression in the substantia nigra, normalized the striatal dopaminergic fibers, and prevented the depletion of dopamine and its metabolites in the striatum. MPTP-treated mice showed cognitive impairments accompanied with significant motor deficiency. Orexin-A improved MPTP-induced impairments in both motor activity and spatial memory. Importantly, orexin-A increased the protein level of brain-derived neurotrophic factor (BDNF) in dopaminergic neurons of the substantia nigra. Furthermore, the protective effects of orexin-A on MPTP parkinsonian mice could be blocked by orexinergic receptor 1 (OX1R) antagonist, SB334867. In another set of experiments with SH-SY5Y dopaminergic cells, orexin-A significantly induced the expression of BDNF in a dose and time-dependent manner. The upregulation of BDNF is mainly concerned with PI3K and PKC signaling pathways via OX1R. The present study demonstrated that orexin-A exerted neuroprotective effects on MPTP parkinsonian mice, which may imply orexin-A as a potential therapeutic target for PD.

## Introduction

Parkinson’s disease is the second most common chronic neurodegenerative disease characterized by progressive loss of dopaminergic neurons in the SNpc (Hornykiewicz and Kish, 1987). The incidence of PD in the population over 55 years old is about 1% and the cardinal symptoms of PD include resting tremor, bradykinesia, muscle rigidity, postural instability, and usually companied with cognitive impairment, mental disorder, and other non-motor symptoms (Meerwaldt and Hovestadt, 1988; Beitz, 2014). The cause of PD is not fully understood, but several factors including gene mutation, oxidative stress, mitochondrial dysfunction, neurotransmitter toxicity, failure of protein homeostasis appear to be associated with the development of PD. The most common treatment for PD is symptom management. The dopamine precursor levodopa is the most widely used clinical drug (Hornykiewicz, 1975), which could only attenuate the symptoms, but fails to halt the progressive degeneration of dopaminergic neurons in the substantia nigra. In recent years, many efforts were devoted to find endogenous neuroprotective mediators to stop or reverse the degenerative changes of dopaminergic neurons in the substantia nigra.

Orexins, consisting of orexin-A and orexin-B, belong to hypothalamic neuropeptides derived from a common precursor named prepro-orexin (Peyron et al., 1998; Sakurai et al., 1998). Two types of specific G-protein-coupled receptors, orexinergic receptor 1 (OX1R) and orexinergic receptor 2 (OX2R), are involved in the functions of orexins. Cell bodies of orexinergic neurons are strictly localized in the lateral hypothalamic/perifornical area (LH/PFA) (Sakurai et al., 1998), but their fibers are widespread in entire brain. Orexinergic receptors are located in many brain structures, such as cortex, hippocampus, amygdala, thalamus, hypothalamus, and basal ganglia (Hervieu et al., 2001). It is known that orexins play important roles in the regulation of sleep, feeding behavior, energy homeostasis, neuroendocrine, and autonomic control. The activity of orexinergic system decreases with aging, which has been implicated in many neurodegenerative disorders (Zhang et al., 2013; Li et al., 2014; Hu et al., 2015; Chieffi et al., 2017a).

In addition, orexinergic systems also play an important role in motor control (Zhang et al., 2011, 2013; Hu et al., 2015; Chieffi et al., 2017a). Most of the central motor control structures are innervated by orexinergic fibers (Hu et al., 2015). More importantly, all the basal ganglia nuclei, including the globus pallidus, the subthalamic nucleus, the substanita nigra, and the striatum are innervated by orexingeric fibers (Cutler et al., 1999; Schmitt et al., 2012; Dell et al., 2013). Numerous studies demonstrated that orexinergic systems are closely correlated with PD (Yasui et al., 2006). It was reported that parkinsonian patients display significant loss of orexinergic neurons in post-mortem exams (Fronczek et al., 2007; Thannickal et al., 2007). Experiments with 6-hydroxydopamine (6-OHDA)-induced rat model of PD revealed that the number of orexinergic neurons in the lateral hypothalamus decreases significantly (Cui et al., 2010). Furthermore, the orexin levels in plasma and cerebrospinal fluid decrease dramatically in parkinsonian patients (Drouot et al., 2003; Fronczek et al., 2007; Thannickal et al., 2007; Baumann et al., 2008). These reports implied the important role of orexinergic systems in PD. Recent studies revealed that orexin-A has neuroprotective effects in cellular models of PD. Orexin-A protects SH-SY5Y cells against 6-OHDA (Esmaeili-Mahani et al., 2013; Pasban-Aliabadi et al., 2017) or MPP^+^ (Feng et al., 2014) induced toxicity. However, little is known about the exact role of orexin-A in the animal models of PD, and the protective mechanisms of orexin-A on the nigral dopaminergic neurons.

Brain-derived neurotrophic factor is a neurotrophin widely expressed in the mammalian brain, and known to have neuroprotective effects on dopaminergic neurons and cognitive processes. Recent studies suggested some functional correlation between orexin-A and BDNF. Both orexin-A and BDNF exert antidepressive-like effect and memory facilitatory action (Chieffi et al., 2017a,b). Parkinsonian patients have low levels of BDNF (Scalzo et al., 2010) and orexin-A (Drouot et al., 2003). Exercise increases the levels of BDNF and orexins (Messina et al., 2016; Chieffi et al., 2017a,c). Numerous studies have demonstrated the potent protective effects of BDNF on dopaminergic neurons in animal parkinsonian models (Tsukahara et al., 1995; Fumagalli et al., 2006; Stahl et al., 2011), and BDNF is considered to be involved in the beneficial effects of exercise in PD (Real et al., 2013; Angelucci et al., 2015). However, studies about the neuroprotective effects of orexins in parkinsonian models are limited and little is known about the possible relationship between orexins and BDNF.

In the present study, we elucidated the effects of orexin-A on MPTP-induced C57BL/6 mice model of PD. In addition, we further investigated the relationship between orexin-A and BDNF in nigral dopaminergic neurons and in SH-SY5Y human dopaminergic neuroblastoma cells.

## Materials and Methods

### Animals

Male C57BL/6 mice at the age of 10 weeks, weighing 22–26 g, were used in this study. Animals were housed under a 12-h light/dark cycle with food and water available. The experiments were performed according to the National Institutes of Health Guide for the Care and Use of Laboratory Animals (NIH Publications No. 8023) and its 1978 revision, and were approved by the Animal Ethics Committee of Qingdao University. All efforts were made to minimize the number of animals used and their suffering.

### Establishment and Administration of MPTP-Induced Mouse Model of PD

**FIGURE 1:**
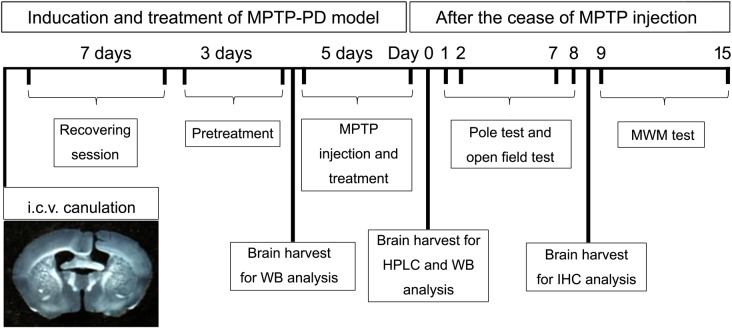
A schematic diagram depicted the experimental design in the present study. Schematic timeline representation for cannula placements of i.c.v. injections, recovery, pretreatment and administration of drugs or vehicles, pole test, open field test, and Morris water maze test. HPLC, high performance liquid chromatography; WB, western blot; MWM, Morris water maze; IHC, immunohistochemistry.

The schematic diagram (Figure 1) depicted the schedules of the animal experiments. Firstly, mice were anesthetized with chloral hydrate (400 mg/kg, i.p.) and placed in a stereotaxic frame. A guide cannula with cannula cap of stainless steel stylet (62003 and 62102, RWD Life Science, China) was implanted into the lateral ventricle on the right side, 0.3 mm posterior, 1.0 mm lateral from the bregma, and 2.2 mm ventral from the skull surface (Paxinos and Franklin, 2004). The cannula was fixed to the skull with stainless steel screws and dental acrylic. After the cannulation, the mice were transferred to individual cages for at least 7 days of recovery. During the first 4 days after surgery, mice received intramuscular injection of penicillin (20,000 U/mouse/day) to prevent infection. After the recovery session, the mice were randomly divided into four groups (*n* = 20–22) and every mouse received both intracerebroventricular (i.c.v.) injection once per day for eight consecutive days and intraperitoneal (i.p.) injection once per day for the last five consecutive days. (1) Control group: mice received saline (i.c.v. and i.p.) injection; (2) MPTP group: mice received saline (i.c.v.) and MPTP (30 mg/kg, i.p.) injection; (3) Orexin-A + MPTP group: mice received orexin-A (300 ng/mouse, i.c.v.) and MPTP (30 mg/kg, i.p.) injection; (4) SB334867 + orexin-A + MPTP group: mice were pretreated with SB334867 (10 mg/kg, i.p.) 30 min before receiving orexin-A (300 ng/mouse, i.c.v.) and MPTP (30 mg/kg, i.p.) injection. In order to prevent infection, all the mice received intramuscular injection of penicillin (20,000 U/mouse/day) during the 8 days of injection. One day after the last injection of MPTP, six mice from each group were killed by cervical vertebra dislocation. The striatum and the substantia nigra were carefully isolated for high performance liquid chromatography (HPLC) analysis and protein extraction respectively. Other mice were subjected to a series of behavioral tests for motor activity (pole test and open field test) and spatial learning and memory (Morris water maze test).

### High Performance Liquid Chromatography With Electrochemical Detection (HPLC-EC) Analysis of DA and Related Metabolites in Striatum

Six mice from each group provided samples for HPLC. The levels of the striatal dopamine (DA) and its metabolites, 3, 4-dihydroxyphenylacetic acid (DOPAC) and homovanillic acid (HVA), were determined by high-performance liquid chromatography equipped with a 2465 electrochemical detector. Briefly, striatum tissues were weighed and then homogenized in 100 μL liquid A (0.4 M perchloric acid). After initial centrifugation (120,000 rpm for 20 min at 4°C), 80 μL of the supernatant was transferred into the new eppendorf tubes, and 40 μL liquid B (20 mM citromalic acid potassium, 300 mM dipotassium phosphate, 2 mM EDTA⋅2Na) was added. After additional centrifugation (120,000 rpm for 20 min at 4°C), 100 μL of the supernatant was assayed for DA and its metabolites, DOPAC and HVA, by HPLC. Separation was achieved on a PEC18 reverse-phase column. The mobile phase consisted of 20 mM citromalic acid, 50 mM sodium caproate, 0.134 mM EDTA⋅2Na, 3.75 mM sodium octane sulfonic acid, and 1 mM disec-butylamine at 5% (v/v) methanol, with the flow-rate of 1 mL/min. A 2465 electrochemical detector (Waters, United States) was operated in screen mode. The results were presented as nanograms per milligram wet weight of brain tissue.

### Behavioral Testing

Open field test (Harms et al., 2008) was used to assay the general locomotor activity. The open field tests were conducted using a square arena (26.5 cm × 26.5 cm × 35.5 cm). In each individual experiment, the mouse was placed into the center of the arena. Behaviors were video-taped for 10 min. Open field tests were performed on day 1 and day 7 after the cease of MPTP injection, and the total distance traveled in each group was calculated as the final results.

Pole test was used to assay movement disorders in mice (Matsuura et al., 1997). The mouse was placed head-upward on the top of a vertical rough-surfaced pole (diameter 1 cm, height 55 cm). The time to turn downward from the top (T-turn time) and to descend to the floor (T-LA time) was measured. Pole tests were conducted on day 2 and day 8 after the cease of MPTP injection.

Spatial learning and memory of the mice were tested by Morris water maze test (Morris, 1984). The circular water maze pool (120 cm in diameter, 30 cm deep) filled with water at 24 ± 1°C was located in a dimly lit room. An escape platform (10 cm in diameter) was submerged about 1 cm below the water surface, in a fixed position in one of the four quadrants (i.e., the target quadrant). Curtains surrounded the pool with distinct cues sticking on (Zhou et al., 2007 and Liu Q. et al., 2018). Mice were handled for 3 min per day over seven consecutive days before training and were trained daily over 7 days. On each training day, mice received four training trials presented in two blocks (inter-block interval 2 h, inter-trial interval 30 s). On each trial, the mice were put into the pool, facing the wall, in one of the six start locations and allowed to search for the platform. The time for the mice to find the platform was recorded as the escape latency, which indicated the learning ability. The animals were allowed to stay on the platform for 30 s if they were able to find the platform within 60 s. However, if an animal did not find the platform within 60 s, the animal was guided to the platform, allowed to remain there for 30 s and the escape latency was recorded as 60 s. On the third, fifth, and seventh days of training session, the spatial memory was evaluated by the probe test. The platform was removed, and the mice were then allowed to swim for 60 s in the pool. The time that the mice swam across the site where the platform was hidden (number of platform-crossing times), the relative time spent in the target quadrant (target quadrant time) and the total distance traveled were automatically recorded with a video connected to a computer. The mean value of escape latency, platform-crossing times, target quadrant time and the total distance traveled in each group were calculated as the final results.

### Double Immunofluorescence Staining

Normal mice were anesthetized by chloral hydrate and were fixed with PFA solution (4% paraformaldehyde in 0.1 M phosphate buffer, pH = 7.4) through heart perfusion. The brains were removed and post-fixed in the PFA solution for 6 h and then cryoprotected in 20 and 30% sucrose at 4°C, respectively. After the dehydration, each brain containing the substantia nigra was cut into 20-μm-thick coronal sections on a freezing microtome (CM 1950, Leica, Germany). Non-specific binding was blocked with 5% BSA in PBS for 1 h. The sections were then incubated overnight with primary antibodies consisting of chicken anti-TH antibody (Abcam, 1:2,000) and rabbit anti-OX1R (Abcam, 1:100) or OX2R (Bioss, 1:200) antibody at 4°C for 36 h in a humidified chamber. Fluorescent isothiocyanate-conjugated goat anti-chicken IgG (Invitrogen, 1:500) and rhodamine-conjugated goat anti-rabbit IgG (Abcam, 1:500) were used as secondary antibodies. Finally, the sections were mounted on polylysine-coated slides and examined using an inverted fluorescent microscope (Axio Observer.A1, Zeiss, Germany). Negative control tests were run without primary or secondary antibodies.

### TH Immunohistochemistry

After the last time of pole test, six mice of each group were sacrificed and perfused with 4% paraformaldehyde solution transcardially. Brains were sectioned into 20-μm-thick coronal sections on a freezing microtome. After PBS rinsing three times (5 min each), the sections were treated with 0.3% H_2_O_2_ for 10 min at room temperature to eliminate the endogenous peroxidase. After PBS rinsing, the sections were blocked in 5% BSA-PBS containing 0.03% TX-100 for 1 h at room temperature. The sections were firstly incubated with the primary antibody against TH (Millipore, 1:1,000) overnight at 4°C, and then washed in PBS and incubated with HRP conjugated goat anti-rabbit IgG, and the color was shown with DAB detection system, according to the instruction of Polink-1 HRP DAB immunochemistry kit (PV6001 and ZLI-9019, Zhongshan Golden Bridge, China). Finally, the sections were mounted on polylysine-coated slides, dehydrated through graded ethanol, and cleared in xylene and finally followed by coverslipping using permount mounting medium. Digital images of TH-ir neurons in substantia nigra were obtained using a bright-field microscope (DM 2500, Leica, Germany). The total number of TH-ir neurons was estimated bilaterally every 4th section through the extent of the substantia nigra of each brain. All sections were coded and examined blind. The survival rate of dopaminergic neurons in the SNpc was determined by comparing the number of TH-ir neurons of the tested group with that of the normal control group. Digital images of TH-ir fibers in striatum sections were obtained with a scanner (V370, Epson, Japan), and the optical density of the striatum was calculated using ImageJ software (NIH, United States).

### Cell Cultures

Human neuroblastoma SH-SY5Y cells were obtained from Shanghai cell bank of Chinese Academy of Sciences. Cells were grown with Dulbecco’s modified Eagle’s medium (DMEM) supplemented with 10% FBS, penicillin (100 U/mL), streptomycin (100 μg/mL), and maintained at 37°C in a 5% CO_2_ atmosphere. Cells were seeded on a 6-well plate and permitted to attach and grow for 24 h. After 24 h, the cells were incubated in serum-free DMEM. Various inhibitors, such as SB334867, LY294002, GF109203X, as selective OX1R antagonist, PI3K antagonist and PKC antagonist, respectively, were added 30 min before starting the experiment with orexin-A.

### Immunoblotting

Samples from cells and animals were lysed in RIPA assay buffer supplemented with a protease inhibitor cocktail, and the protein concentration was determined using BCA protein assay kit (Beyotime, China). Equal amounts of proteins were loaded and separated by 10% SDS-PAGE or 12% SDS-PAGE. The proteins were transferred to PVDF membranes (Millipore, United States), and each membrane was blocked and then incubated with primary antibody at 4°C. After washing, the blots were incubated with HRP-conjugated secondary antibodies for 2 h. The staining was visualized by ECL detection reagents (Millipore, United States). Digital images were obtained using an imaging system (BioSpectrum 810, VUP, United States). The primary antibodies used were anti-TH (Millipore, 1:2,000), anti-OX1R (Abcam, 1:1,000), anti-PI3K (Bioss, 1:1,000), and anti-BDNF (Bioss, 1:1,000). Anti-mouse HRP (Abcam, 1:10,000) and anti-rabbit HRP (Sigma, 1:8,000) were used as secondary antibodies. Anti-β-actin (Bioss, 1:10,000) served as a loading control. The optical density and area of the protein band were calculated using ImageJ software (NIH, United States) from band densitometry. And the density values were expressed as tested proteins/β-actin ratio for each sample.

### Drugs and Statistical Analysis

Unless otherwise stated, all chemicals were purchased from Sigma Chemical, Co. (St. Louis. MO, United States). Orexin-A and SB334867 were purchased from Tocris (Bristol, United Kingdom). LY294002 and GF109203X were purchased from ApexBio (Boston, MA, United States). Results shown in the figures were representative of at least three independent experiments. All values in the figures and the text were expressed as mean ± SEM. Data analysis was performed using IBM SPSS Statistics 22.0 software and Prism 5.0. The results were analyzed by one-way or two-way analysis of variance followed by a Bonferroni *post hoc* test for multiple comparisons. *P* < 0.05 was considered statistically significant.

## Results

### Both of the Orexinergic Receptor Subtypes, OX1R and OX2R, Were Expressed in the Substantia Nigra Region

**FIGURE 2:**
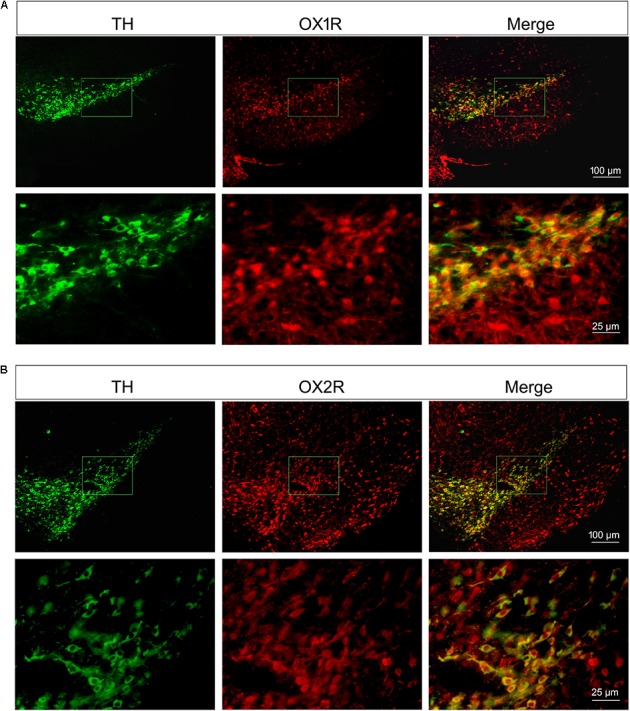
Orexinergic receptor subtypes OX1R and OX2R co-localized with TH in the substantia nigra. Both of the OX1R **(A)** and OX2R **(B)** were expressed in the substantia nigra region and VTA. Double-labeling immunofluorescence showed the co-localization of TH (green in **A,B**) and OX1R (red in **A**) or OXR2 (red in **B**) on the dopaminergic neurons in the SNpc.

As shown in Figure 2, both of the OX1R and OX2R were expressed in the substantia nigra region and ventral tegmental area (VTA). TH was selectively expressed in the dopaminergic neurons. Double-labeling immunofluorescence showed that both OX1R (red, Figure 2A) and OX2R (red, Figure 2B) were co-localized with TH (green, Figures 2A,B) on the dopaminergic neurons in the SNpc. The present studies demonstrated that nigral dopaminergic neurons expressed two orexinergic receptor subtypes.

### Orexin-A Reversed MPTP-Induced Motor Impairments via OX1R

**FIGURE 3:**
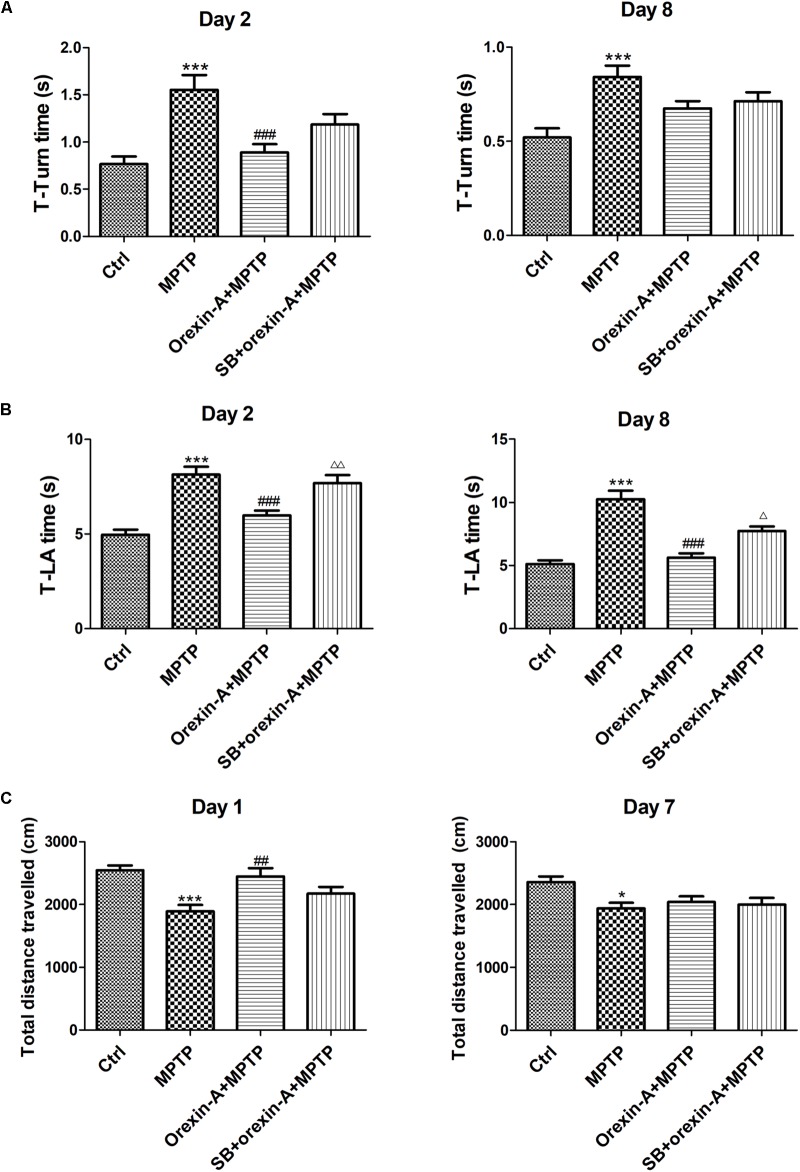
Effects of orexin-A on the motor activity impairments induced by MPTP intoxication in pole test **(A,B)** and open field test **(C)**. T-turn time **(A)** and T-LA time **(B)** in pole test were recorded on day 2 and day 8 after the cease of MPTP treatment. **(C)** Total distance traveled in 10 min-duration open field test was recorded on day 1 and day 7 after the cease of MPTP treatment. Values were mean ± SEM, *n* = 12–14. ^∗^*P* < 0.05, ^∗∗∗^*P* < 0.001 vs. control; ^##^*P* < 0.01, ^###^*P* < 0.001 vs. MPTP; ^Δ^ *P* < 0.05, ^ΔΔ^ *P* < 0.01 vs. orexin-A + MPTP. SB: SB334867.

The motor function of all the experimental groups was assessed by pole test and open field test on different days. In pole test, both of T-turn time (Figure 3A) and T-LA time (Figure 3B) were recorded on day 2 and day 8, separately. MPTP-treated mice showed significant motor disorders as indicated by an increase in T-turn time on day 2 (control: 0.77 ± 0.08 s; MPTP: 1.56 ± 0.15 s; *n* = 14, *P* < 0.001) and on day 8 (control: 0.52 ± 0.05 s; MPTP: 0.84 ± 0.06 s; *n* = 12, *P* < 0.001). However, orexin-A treatment significantly reduced the T-turn time compared with MPTP group on day 2 (orexin-A + MPTP: 0.89 ± 0.09 s; *n* = 14, *P* < 0.001), but not significant on day 8 (orexin-A + MPTP: 0.67 ± 0.04 s; *n* = 12, *P* > 0.05). Furthermore, MPTP-treated mice also showed an extended T-LA time on both day 2 (control: 4.95 ± 0.28 s; MPTP: 8.14 ± 0.42 s; *n* = 14, *P* < 0.001) and day 8 (control: 5.12 ± 0.30 s; MPTP: 10.26 ± 0.67 s; *n* = 12, *P* < 0.001). Orexin-A treatment significantly reduced the T-LA time compared with MPTP group on day 2 (orexin-A + MPTP: 5.97 ± 0.26 s; *n* = 14, *P* < 0.001) and day 8 (orexin-A + MPTP: 5.62 ± 0.37 s; *n* = 12, *P* < 0.001). OX1R selective antagonist SB334867 significantly attenuated the protective effect of orexin-A on both day 2 (SB334867 + orexin-A + MPTP: 7.68 ± 0.43 s; *n* = 14, *P* < 0.01) and day 8 (SB334867 + orexin-A + MPTP: 7.73 ± 0.38 s; *n* = 12, *P* < 0.05).

Open field test was conducted on day 1 and day 7, in which the total distance traveled in the arena was calculated to assess the spontaneous activity of the mice (Figure 3C). The total distance traveled in MPTP group reduced on day 1 (control: 2545 ± 76.74 cm; MPTP: 1891 ± 99.31 cm; *n* = 14, *P* < 0.001) and day 7 (control: 2358 ± 87.99 cm; MPTP: 1937 ± 91.12 cm; *n* = 12, *P* < 0.05). MPTP-induced reduction of total distance traveled was significantly reversed by orexin-A treatment on day 1 (orexin-A + MPTP: 2444 ± 133.7 cm, *n* = 14, *P* < 0.01). SB334867 had some tendency to antagonize the effect of orexin-A on the same day (SB334867 + orexin-A + MPTP: 2171 ± 111.1 cm, *n* = 14, *P* = 0.128), although there was no significant difference. However, 7 days after last injection of MPTP, the total distance showed no difference between MPTP group and orexin-A treatment group. The results suggested that orexin-A-induced improvement in spontaneous physical activity of MPTP parkinsonian mice was temporary and not sustained longterm.

### Orexin-A Improved MPTP-Induced Impairments in Spatial Learning and Memory

**FIGURE 4:**
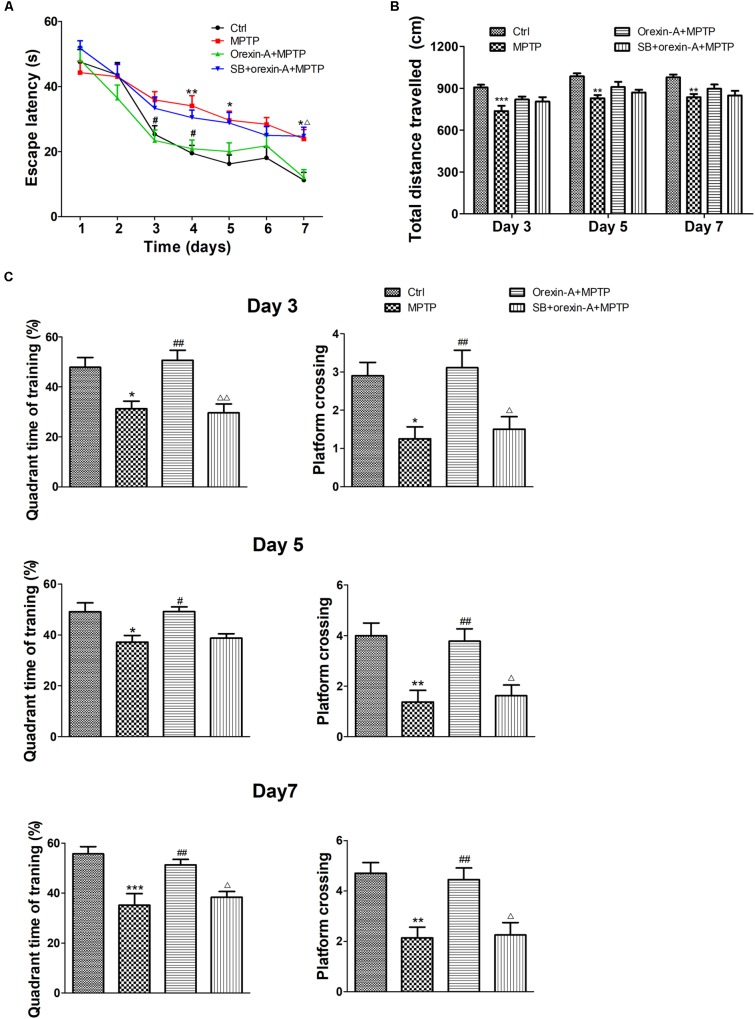
Effects of orexin-A on MPTP-induced impairments in spatial learning and memory. **(A)** Effects of orexin-A on escape latency at the training session. **(B)** Total distance traveled in the probe test on day 3, day 5, and day 7 of training. **(C)** Two hours after the second training trial, retention of memory was tested in the 60 s probe trial on day 3, day 5, and day 7 of training session, separately. Each value represented mean ± SEM, *n* = 8–10. ^∗^*P* < 0.05, ^∗∗^*P* < 0.01, ^∗∗∗^*P* < 0.001 vs. control; ^#^*P* < 0.05, ^##^*P* < 0.01 vs. MPTP; ^Δ^ *P* < 0.05, ^ΔΔ^ *P* < 0.01 vs. orexin-A + MPTP. SB: SB334867.

To determine whether impaired learning and memory of MPTP-intoxicated mice could be reversed by orexin-A, Morris water maze test was conducted on 9 days after the cease of MPTP injection. The latency of the animal to reach the hidden platform over the seven training days was shown in Figure 4A. MPTP-treated mice took longer time to find the hidden platform than the mice of control group. There was a significant difference in escape latency between MPTP group and control group on the fourth day (control: 19.45 ± 2.46 s, *n* = 10; MPTP: 34.03 ± 3.13 s, *n* = 8; *P* < 0.01), fifth day (control: 16.23 ± 2.73 s, *n* = 10; MPTP: 29.63 ± 2.75 s, *n* = 8; *P* < 0.05) and seventh day (control: 11.23 ± 2.34 s, *n* = 10; MPTP: 23.88 ± 2.90 s, *n* = 8; *P* < 0.05) of the training session. Orexin-A treatment obviously reduced the escape latency to a comparable level of control group, a statistical difference was found between orexin-A + MPTP group and MPTP group on the third day (MPTP: 35.84 ± 2.59 s, *n* = 8; orexin-A + MPTP: 23.39 ± 3.23 s, *n* = 9; *P* < 0.05) and fourth day (MPTP: 34.03 ± 3.13 s, *n* = 8; orexin-A + MPTP: 20.86 ± 2.68 s, *n* = 9; *P* < 0.05) of training. SB334867 obviously attenuated the improvement induced by orexin-A on the seventh day (orexin-A + MPTP: 12.08 ± 2.37 s, *n* = 9; SB334867 + orexin-A + MPTP: 24.72 ± 2.77, *n* = 8; *P* < 0.05) and had some tendency to attenuate the improvement on the third day (orexin-A + MPTP: 23.39 ± 3.23 s, *n* = 9; SB334867 + orexin-A + MPTP: 33.31 ± 3.47, *n* = 8; *P* = 0.170) and fourth day (orexin-A + MPTP: 20.86 ± 2.68 s, *n* = 9; SB334867 + orexin-A + MPTP: 30.44 ± 2.26, *n* = 8; *P* = 0.105). The results suggested that orexin-A treatment restored the spatial learning and memory impairment induced by MPTP, and the protective effect of orexin-A could be partially eliminated by SB334867.

In order to determine whether the improvement of orexin-A in escape latency is primarily due to the difference in swimming ability, the total distance traveled in probe test was analyzed as an indicator of motor activity (Figure 4B). The results showed that the total distance traveled in MPTP group reduced compared with control group in all test days. However, orexin-A treatment only induced weak recovery of the value, but there was no significant difference. These results indicated that orexin-A treatment improved the spatial learning and memory impairment in MPTP-intoxicated mice, which was not completely due to its positive effect on motor activity.

Similar protective effects of orexin-A on spatial memory ability were observed at the probe test on the third, fifth, and seventh days of training (Figure 4C). Numbers of platform crossing and target quadrant time of MPTP group significantly decreased compared with control group in all the probe tests. Orexin-A treatment significantly elevated the two parameters to a comparable level of control. The protective effect of orexin-A in probe test could be antagonized by SB334867. These results suggested that orexin-A significantly improved MPTP-induced impairments in spatial learning and memory mainly through OX1R.

### Orexin-A Protected Dopaminergic Neurons Against MPTP-Induced Neurotoxicity

#### Orexin-A Attenuated the Depletion of Dopamine in the Striatum

**FIGURE 5:**
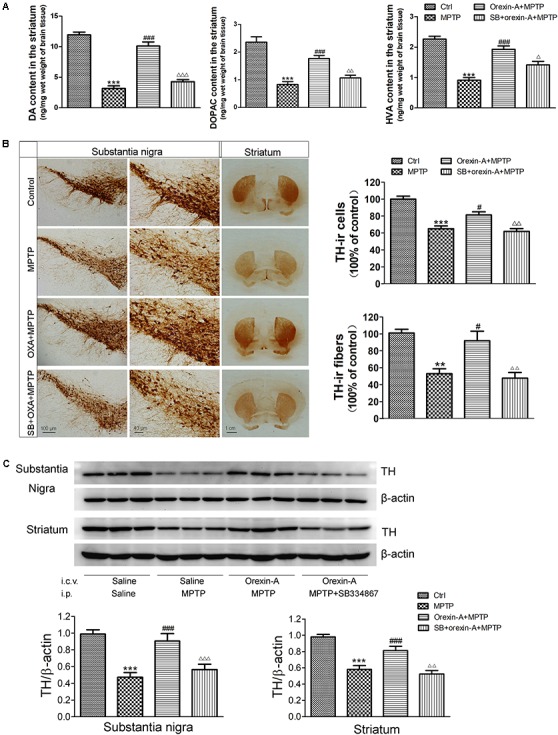
Effects of orexin-A on DA levels and TH protein levels in the substantia nigra and striatum of MPTP-intoxicated mice. **(A)** HPLC analysis showing the striatal DA, DOPAC, and HVA levels in different groups (*n* = 6). **(B)** Immunohistochemistry showing the dopaminergic neurons in the substantia nigra and the dopaminergic fibers in the striatum of different groups. Photomicrographs of representative substantia nigra and striatal sections stained with TH antibodies. Quantitative analysis of TH-ir neurons in the substantia nigra (*n* = 6) and TH-ir fibers in the striatum (*n* = 4). **(C)** Immunoblot analysis showing the TH protein levels in the substantia nigra and striatum of different groups. Average protein level was quantified as ratios between TH and β-actin (*n* = 6). ^∗∗^*P* < 0.01, ^∗∗∗^*P* < 0.001 vs. control; ^#^*P* < 0.05, ^###^*P* < 0.001 vs. MPTP; ^Δ^ *P* < 0.05, ^ΔΔ^ *P* < 0.01, ^ΔΔΔ^ *P* < 0.001 vs. orexin-A + MPTP. OXA: orexin-A, SB: SB334867.

High performance liquid chromatography analysis (Figure 5A) revealed that systemic injection of MPTP resulted in a significant depletion of DA and its metabolites, DOPAC and HVA, in the striatum. The DA levels of MPTP group reduced by 73.60% of control group (control: 11.93 ± 0.48 ng/mg; MPTP: 3.15 ± 0.44 ng/mg; *n* = 6, *P* < 0.001). Orexin-A-treatment significantly restored the level of DA concentration to 84.74% of control group (orexin-A + MPTP: 10.11 ± 0.68 ng/mg; *n* = 6, *P* < 0.001), and the protective effect of orexin-A could be antagonized by SB334867 (SB334867 + orexin-A + MPTP: 4.27 ± 0.34 ng/mg; *n* = 6, *P* < 0.001). Similar results were also observed in the levels of DOPAC and HVA. These data suggested that orexin-A conferred a significant protective effect against MPTP-induced DA depletion.

#### Orexin-A Attenuated the Reduction of Dopaminergic Neurons in the Substantia Nigra and Restored the Reduction of Dopaminergic Fibers in the Striatum

1-Methyl-4-phenyl-1, 2, 3, 6- tetrahydropyridine-treated mice showed a significant loss of TH-immunoreactive (TH-ir) neurons in the substantia nigra (Figure 5B). The survival ratio of nigral TH-ir neurons in MPTP mouse model decreased to 65.0 ± 3.4% of control group (*n* = 6, *P* < 0.001). Orexin-A treatment exerted significant protective effects on dopaminergic neurons against MPTP intoxication. Orexin-A treatment preserved as many as 81.3 ± 3.6% of control group, which was significantly different from that of MPTP group (*n* = 6, *P* < 0.05). SB334867 abolished the neuroprotective effect of orexin-A (*n* = 6, *P* < 0.01). Immunohistochemistry staining of the striatum showed that the number of TH-ir fibers of MPTP treated mice markedly reduced to 52.9 ± 5.8% of control group (*n* = 4, *P* < 0.01). Orexin-A treatment restored the TH-ir fibers up to 92.0 ± 11.1% of the control group (*n* = 4, *P* < 0.05), and the protective effect of orexin-A could be antagonized by SB334867 (*n* = 4, *P* < 0.01). The results from immunoblot analysis (Figure 5C) were in accordance with that from immunohistochemistry. These data demonstrated that orexin-A protected the dopaminergic neurons in the substantia nigra and restored the reduction of TH-ir fibers in the striatum induced by MPTP intoxication, and the protective effect could be antagonized by OX1R antagonist SB334867.

### Orexin-A Increased the Protein Expression of BDNF in Nigral Dopaminergic Neurons

**FIGURE 6:**
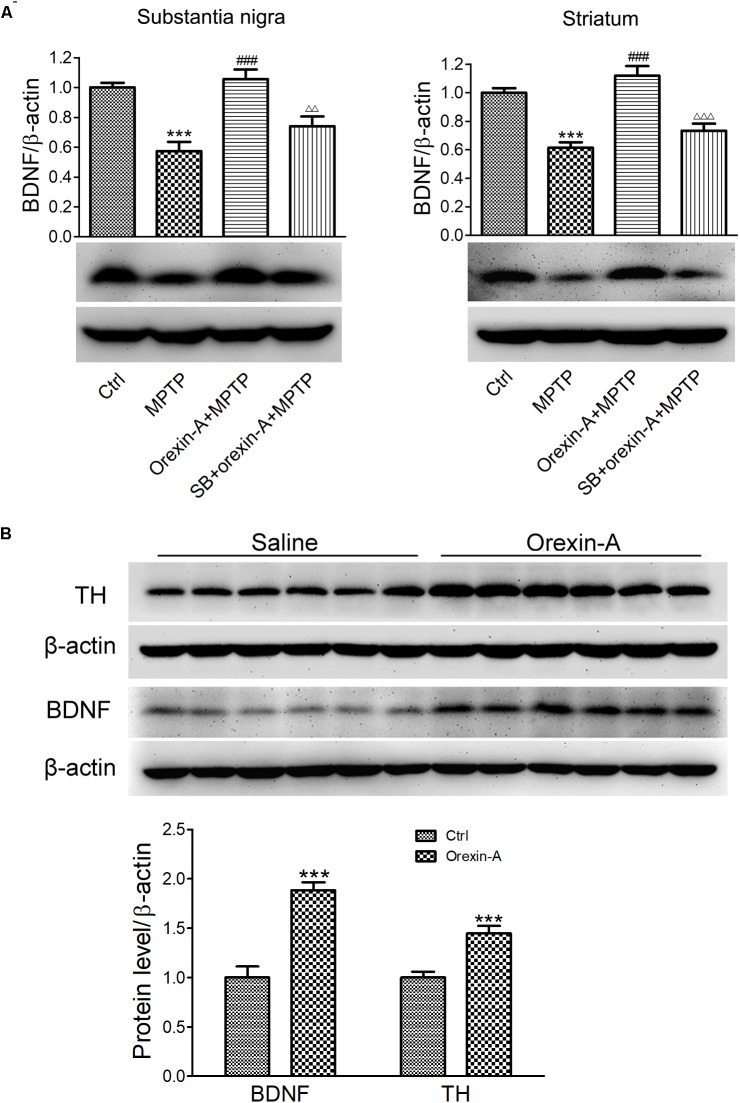
Effects of orexin-A on the protein expression of BDNF. **(A)** Immunoblot analysis showing the protein expression of BDNF in the substantia nigra and striatum of MPTP parkinsonian mice. Average protein level was quantified as ratios between BDNF and β-actin. **(B)** Immunoblot analysis showing the effects of orexin-A on protein expression of BDNF in the striatum after 3 days of orexin-A injection in normal mice. Average protein level was quantified as ratios between BDNF or TH and β-actin. Each value represented mean ± SEM, *n* = 6. ^∗∗∗^*P* < 0.001 vs. control; ^###^*P* < 0.001 vs. MPTP; ^ΔΔ^ *P* < 0.01, ^ΔΔΔ^ *P* < 0.001 vs. orexin-A + MPTP. SB: SB334867.

Brain-derived neurotrophic factor is a principal regulator of axonal growth and connectivity, neuronal differentiation, survival, and synaptic plasticity in mammals. BDNF has been demonstrated to be linked to neurodegenerative diseases due to the neuroprotective effects. Next, we determined whether BDNF is involved in orexin-A-induced protective effects in MPTP neurotoxicity. The protein levels of BDNF in the substantia nigra and striatum were detected by immunoblot analysis (Figure 6A). The results showed that MPTP treatment reduced the BDNF protein level to 57.4 ± 6.2% in the substantia nigra compared with the control group (*n* = 6, *P* < 0.001). Furthermore, orexin-A treatment restored the protein expression of BDNF up to 105.7 ± 6.4% of the control level (*n* = 6, *P* < 0.001), and SB334867 reduced the effect of orexin-A to 74.1 ± 6.5% of the control group (*n* = 6, *P* < 0.01). Similar results were observed from the striatum. Surprisingly, striatal BDNF in orexin-A + MPTP group elevated to 111.9 ± 6.7% of control group, although the increase was not significant (*n* = 6, *P* > 0.05). The results indicated that BDNF might be involved in the neuroprotective effects of orexin-A in the dopaminergic neurons.

To determine whether orexin-A alone could induce the expression of BDNF in the striatum, 12 normal mice were divided into two groups, receiving orexin-A (300 ng/day) or vehicle (normal saline) i.c.v. injection for three consecutive days. The protein level of BDNF in the striatum was detected by immunoblot analysis (Figure 6B). The results showed that orexin-A significantly increased the protein level of BDNF in the striatum by 88.3 ± 8.1% (*n* = 6, *P* < 0.001). Furthermore, the protein level of TH increased by 44.6 ± 7.6% (*n* = 6, *P* < 0.001). These results suggested that orexin-A significantly induced the upregulation of BDNF and TH in the striatum of normal mice.

### Orexin-A Increased the Expression of BDNF in SH-SY5Y Human Dopaminergic Neuroblastoma Cells

**FIGURE 7:**
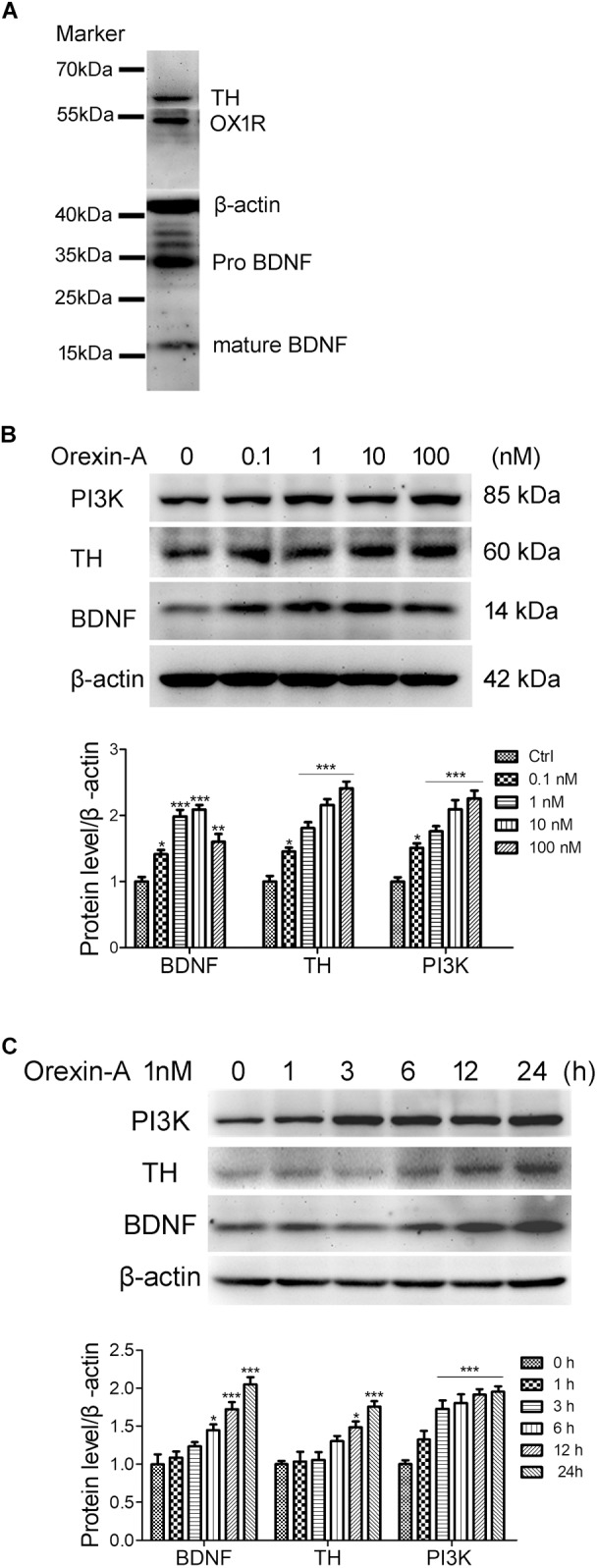
Orexin-A increased the expression of TH and BDNF in SH-SY5Y human dopaminergic neuroblastoma cells. **(A)** Immunoblot analysis revealed the expression of OX1R, TH, and BDNF in SH-SY5Y cells. Original images of the blots are shown in Supplementary Figures S1–S5. **(B)** Protein expression of PI3K, TH, and BDNF in SH-SY5Y cells treated with orexin-A at various concentrations for 24 h. **(C)** Protein expression of PI3K, TH, and BDNF in SH-SY5Y cells treated with 1 nM orexin-A for different time periods. Average protein level was quantified as ratios between BDNF/TH/PI3K and β-actin. Data were presented as mean ± SEM of four independent experiments. ^∗^*P* < 0.05, ^∗∗^*P* < 0.01, ^∗∗∗^*P* < 0.001 vs. control.

Human neuroblastoma SH-SY5Y is a dopaminergic neuronal cell line, which is widely used as an *in vitro* model for the study of PD. In this study, we used the SH-SY5Y cells to investigate the effects of orexin-A on the expression of BDNF and TH. Immunoblot analysis showed that SH-SY5Y cells expressed the proteins of OX1R, BDNF, and TH (Figure 7A). To observe the dose-dependent effect of orexin-A, SH-SY5Y cells were treated with orexin-A of different concentration (0.1, 1, 10, 100 nM) for 24 h (Figure 7B). Immunoblot analysis showed that at the concentration of 0.1 to 100 nM, orexin-A increased the expression of PI3K and TH in a dose-dependent manner. More importantly, orexin-A started to increase the expression of BDNF at 0.1 nM by 41.3 ± 6.5% (*n* = 4, *P* < 0.05), and the most significant BDNF expression was induced at the dose of 1 and 10 nM by two times of the control (*n* = 4, *P* < 0.001). However, when the dose of orexin-A increased to 100 nM, the protein level of BDNF had no further increase. Therefore, orexin-A at 1 nM was selected for the further experiments.

To observe the time-dependent effect of orexin-A, SH-SY5Y cells were treated with 1 nM orexin-A for 1, 3, 6, 12, and 24 h. The results showed that orexin-A increased the expression of BDNF, TH, and PI3K in a time-dependent manner (Figure 7C). The expression of PI3K rapidly increased by 72.6 ± 11.3% as soon as 3 h after orexin-A treatment (*n* = 4, *P* < 0.001), which maintained at high levels in the following 24 h. The protein level of BDNF began to increase at 6 h, and the increased rate was 44.8 ± 7.5% (*n* = 4, *P* < 0.05). The protein level of TH significantly increased at 12 h by 48.5 ± 7.9% (*n* = 4, *P* < 0.05). All of the three proteins reached significantly high levels at 24 h after orexin-A treatment. These results suggested that orexin-A induced the protein expression of BDNF and TH in a time and dose-dependent manner in the SH-SY5Y cells. The OX1R-mediated PI3K signaling pathway might be involved in orexin-A-induced effects.

### PI3K and PKC Pathways Are Involved in Orexin-A-Induced BDNF Expression in Dopaminergic Neuroblastoma SH-SY5Y Cells

**FIGURE 8:**
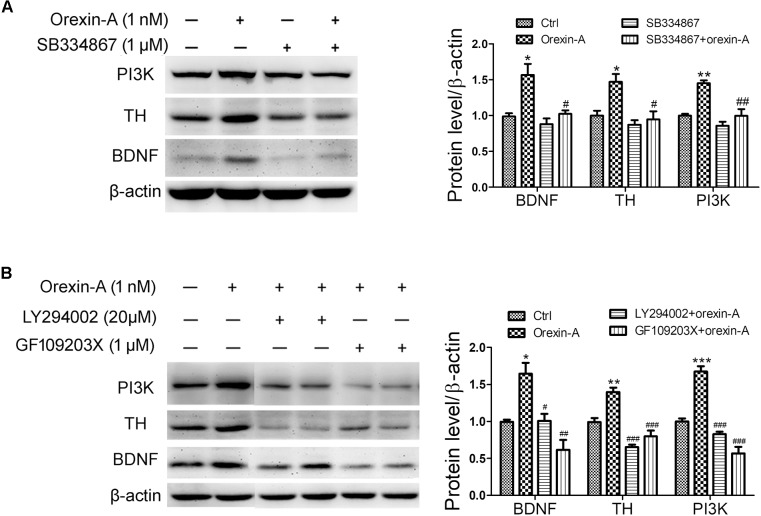
Effects of signaling pathway inhibitors on the expression of BDNF induced by orexin-A in SH-SY5Y cells. **(A)** Effects of OX1R antagonist (SB334867) on the protein levels of PI3K, TH, and BDNF induced by orexin-A. ^∗^*P* < 0.05, ^∗∗^*P* < 0.01 vs. control; ^#^*P* < 0.05, ^##^*P* < 0.01 vs. orexin-A. **(B)** Effects of PI3K inhibitor (LY294002) and PKC inhibitor (GF109203X) on the upregulation of TH and BDNF induced by orexin-A. Average protein level was quantified as ratios between BDNF/TH/PI3K and β-actin. Data were presented as mean ± SEM of three independent experiments. ^∗^*P* < 0.05, ^∗∗^*P* < 0.01, ^∗∗∗^*P* < 0.001 vs. control; ^#^*P* < 0.05, ^##^*P* < 0.01, ^###^*P* < 0.001 vs. orexin-A. Original images of the blots are shown in Supplementary Figures S6–S10.

To determine the possible signaling pathways involved in the upregulation of BDNF and TH induced by orexin-A, OX1R antagonist SB334867, PI3K inhibitor LY294002 and PKC inhibitor GF109203X were employed in SH-SY5Y cells. Application of SB334867 (1 μM) completely blocked the protein upregulation of PI3K, BDNF, and TH induced by orexin-A (1 nM) (Figure 8A). Furthermore, both LY294002 (20 μM) and GF109203X (1 μM) abolished orexin-A-induced upregulation of BDNF and TH (Figure 8B). Therefore, the present experiments demonstrated that the orexin-A-induced expression of BDNF and TH in SH-SY5Y cells was mainly through the OX1R-mediated PI3K and PKC pathways.

## Discussion

Recent studies indicated that central orexin systems are associated with PD. The present study revealed that i.c.v. administration of orexin-A exerted neuroprotective effects on MPTP-induced C57BL/6 mice model of PD. Orexin-A treatment significantly protected the dopaminergic neurons in SNpc and improved the motor and cognitive impairments induced by MPTP intoxication. We demonstrated that orexin-A increased the protein expression of BDNF in the dopaminergic neurons of substantia nigra in both MPTP-treated mice and normal mice. The protective effects of orexin-A were mainly medicated by OX1R.

### Both of the Orexinergic Receptor Subtypes OX1R and OX2R Were Expressed Abundantly on the Dopaminergic Neurons of SNpc

It was reported that moderate concentration of orexin-A is detected in the substantia nigra (Peyron et al., 1998), and the orexin fibers are observed in close proximity to TH-positive dendrites of the neurons in SNpc (Bensaid et al., 2015). Some researchers reported that both OX1R and OX2R are expressed in the substantia nigra (Hervieu et al., 2001; Marcus et al., 2001; Cluderay et al., 2002). However, *in vitro* whole cell patch-clamp recordings showed that dopaminergic neurons in the SNpc are unaffected by orexins (Korotkova et al., 2002). Therefore, whether functional orexinergic receptors are expressed on the dopaminergic neurons is controversial. In present morphological study, double immunofluorescence staining demonstrated that both of the OX1R and OX2R were expressed abundantly on the dopaminergic neurons in SNpc, suggesting a possible direct impact of orexins on nigral DA neurons. In addition, both OX1R and OX2R were also expressed in the substantia nigra pars reticulata and VTA, and no clear difference in the density of OX1R or OX2R was observed between VTA and substantia nigra.

### Orexin-A Significantly Protected the Dopaminergic Neurons in SNpc and Improved the Motor and Cognitive Impairments Induced by MPTP Intoxication

It is well-known that motor and cognitive impairments occur in PD. In present study, MPTP-induced parkinsonian mice showed obvious impairments in both locomotion and cognition. Orexin-A treatment produced dramatically protective effects on both behavioral performance and pathological changes in MPTP-treated mice. Behavioral studies showed that orexin-A treatment improved the performance of MPTP-treated mice in pole test, open field test (Figure 3) and Morris water maze test (Figure 4). These findings indicated profound neuroprotective effects of orexin-A on motor coordination, spontaneous locomotion and spatial memory in MPTP parkinsonian mice. Furthermore, HPLC analysis showed that orexin-A prevented the depletion of DA and its metabolites in the striatum of MPTP mice. Moreover, the present immunohistochemistry provided morphological evidence for orexin-A-induced neuroprotection through a dramatic rescue of the dopaminergic neuronal death (Figure 5).

Accumulating evidence suggested that a major effect of orexin-A signaling is to increase the locomotor activity. Early studies demonstrated that i.c.v. administration of orexin-A or microinjection of orexin-A into certain nucleus, such as hypothalamic paraventricular nucleus, rostral lateral hypothalamic area and nucleus accumbens, locus coeruleus, dorsal raphe nucleus, tuberomammillary nucleus and substantia nigra, apparently elevates the spontaneous physical activity (Kotz et al., 2002, 2006; Kiwaki et al., 2004; Thorpe and Kotz, 2005; Teske et al., 2013). In addition to the neuroprotective effects of orexin-A on dopaminergic neurons, the excitatory effects of orexin-A may also participate in the improvement of motor behavior. Electrophysiological studies showed that orexins increase the firing rate of neurons participating in motor control. For example, orexins excite the neurons of cerebellar interpositus nucleus (Yu et al., 2010) and globus pallidus internus in rat (Gao et al., 2017). Our previous study also revealed that orexins increase the firing rate of the subthalamic neurons (Sheng et al., 2018) and globus pallidus neurons in rats (Xue et al., 2016). All these findings indicate that orexin-A might be a potential target for the treatment of parkinsonian motor deficiency. In present study, we demonstrated that chronic i.c.v. administration of orexin-A improved the motor activity of MPTP parkinsonian mice. Orexin-A treated mice showed a significant improvement in pole test on both day 2 and day 8 (Figures 3A,B). However, only temporary improvement (on day 1) in physical activity was observed in open field test after the treatment of orexin-A (Figure 3C). We interpreted that the increased spontaneous physical activity of orexin-A treatment on day 1 might be partially associated with the direct excitatory effect of orexin-A on some motor function associated central neurons, while the prolonged motor improvement is probably due to its neuroprotection on dopaminergic neurons in SNpc. Consistently, our recent published work confirmed that orexin-A increases the spontaneous firing rate of dopaminergic neurons in the SNpc (Liu C. et al., 2018).

Cognitive abnormalities are common symptoms in parkinsonian patients (Angelucci et al., 2015; Wang et al., 2016) and animal models (De Leonibus et al., 2007). Management of cognitive problems of parkinsonian patients may help to improve their quality of life. Recent studies have suggested that orexins are involved in learning and memory processes. Orexin-deficient mice exhibit impairments in spatial working memory (Dang et al., 2018) and two-way active avoidance memory (Mavanji et al., 2017), while orexin-A replacement restores the cognitive impairment (Mavanji et al., 2017). Furthermore, orexin-A enhances hippocampal neurogenesis and attenuates memory deficits in rats with hippocampal atrophy induced by pentylenetetrazol (Zhao et al., 2014). Orexin-A also improves memory deficiency in a mouse model of Alzheimer’s disease (Jaeger et al., 2002). Importantly, intravenous or intranasal administration of orexin-A to non-human primates rescues cognitive impairments due to sleep-deprivation (Deadwyler et al., 2007). The protection of orexin-A on cognition is considered to be relevant with enhancing long-term potentiation (Wayner et al., 2004; Akbari et al., 2008, 2011), and positively related to the neurogenesis and synaptic plasticity in hippocampus (Stoyanova et al., 2010, 2012; Yang et al., 2013; Zhao et al., 2014). In present study, we demonstrated that chronic i.c.v. administration of orexin-A for 8 days significantly improved the performance of MPTP-treated mice in Morris water maze test (Figure 4). It should be mentioned that the Morris water maze test was conducted on day 9 to day 15 after the treatment. Therefore, it is reasonable to assume that the enhanced neurogenesis and synaptic plasticity in hippocampus might account for the improvement of orexin-A on the cognition of parkinsonian mice. However, the direct excitatory effects of orexin-A on hippocampal neurons (Chen et al., 2017) may also be involved in the cognitive improvement in the present study.

Growing evidence indicated that the substantia nigra is involved in learning and memory processing. Experiments with foot shock test showed that post-trial electrical stimulation in SNpc disrupts retention performance of rats (Routtenberg and Holzman, 1973). A rat model of PD induced by intranigral administration of MPTP shows obvious memory disabilities (Da Cunha et al., 2002). Furthermore, post-training infusion of lidocaine into the substantia nigra produces marked memory deficits (Salado-Castillo et al., 2011). Recent study revealed that the substantia nigra participates in the hippocampal-basal ganglia-frontal cortex loop, and accounts for the encoding of information into long-term declarative memory (Kaminski et al., 2018). These studies collectively indicated that the substantia nigra is an important brain structure involved in memory processing. Thus, we assumed that the improvement of orexin-A on cognition of MPTP parkinsonian mice might be partially due to its protection on dopamingeric neurons in SNpc.

### Orexin-A Increased the Protein Expression of BDNF in the Dopaminergic Neurons of Substantia Nigra in MPTP-Treated Mice and Normal Mice

Brain-derived neurotrophic factor has potent effects on both survival and neurite outgrowth from nigral dopaminergic neurons, and low levels of BDNF might be correlated with PD. Previous studies showed that the mRNA expression of BDNF reduces in the substantia nigra of parkinsonian patients (Howells et al., 2000), and the inhibition of BDNF expression leads to the loss of nigral dopaminergic neurons (Porritt et al., 2005; Fumagalli et al., 2006). Chronic deprivation of BDNF-TrkB signaling pathway leads to selective late-onset nigrostriatal dopaminergic degeneration (Baydyuk et al., 2011a,b). In addition, epidemiological studies showed that low serum levels of BDNF are associated with the motor (Scalzo et al., 2010) and cognitive impairments (Angelucci et al., 2015; Khalil et al., 2016; Wang et al., 2016) in parkinsonian patients. Thus, novel drugs enhancing the biosynthesis of BDNF are considered to be putative therapeutics to prevent the ongoing neurodegenerative of PD.

It is known that orexin-A plays an important role in the regulation of neuroendocrine in the organism (Lopez et al., 2010). There are complex connections between orexins and other hormones, such as corticotrophin-releasing hormone (Lopez et al., 2010), growth hormones (Lopez et al., 2004; Seoane et al., 2004) and neuropeptide Y (Jaszberenyi et al., 2001; Lopez et al., 2002). Previous *in vitro* studies with SH-SY5Y cells showed that orexin-A at the dose of 50 nM induces the expression of vascular endothelial growth factor and erythropoietin (Feng et al., 2014). Moreover, it was reported that hypothalamic BDNF is essential for the orexin-A-induced protection on glucose intolerance and neuronal damage in the cerebral ischemic perfusion mice model (Harada et al., 2013). As orexin-A and BDNF have similar functions in many physiological processes and diseases (Chieffi et al., 2017a,b), and nigral dopaminergic neurons have the ability to produce BDNF for neuroprotection via either autocrine or paracrine mechanisms (Chun et al., 2000; Howells et al., 2000), we hypothesized that the present orexin-A-induced neuroprotection may be associated with the production of BDNF. However there is no study revealing that orexin-A modulates the expression of BDNF in dopaminergic neurons. It was reported that both orexin-A and orexin-B significantly increase the mRNA expression of neurotrophin-3 (NT3) at 10 nM in rat primary cortical neurons. Orexin-B increases the mRNA level of BDNF in primary cortical neuron by twofold at a rather high concentration of 1 μM for 6 days of incubation, while orexin-A does not have such an effect even at the same condition (Yamada et al., 2009). These findings suggest that orexin-A may be a potent inducer for NT3, but not for BDNF in the cerebral cortex. The present study demonstrated that orexin-A increased the expression of BDNF in dopaminergic neurons both *in vivo* and *in vitro*. Orexin-A treatment restored the reduced protein level of BDNF in MPTP-treated mice, and the protein levels of BDNF in the substantia nigra and striatum were even higher than that in control group (Figure 6A). These results suggested that the restoration of BDNF induced by orexin-A treatment is not merely owing to the protection on dopaminergic neurons, but also due to the induction of BDNF in the survival neurons. Furthermore, in normal mice, i.c.v. microinjection with orexin-A also increased the protein levels of BDNF and TH in the striatum (Figure 6B), which was further confirmed in SH-SY5Y human dopaminergic neuroblastoma cells (Figure 7B). Orexin-A induced the BDNF expression at a very low concentration of 0.1 nM in SH-SY5Y cells. These data indicated that orexin-A might be a special and potent inducer of BDNF in the dopaminergic neurons.

Orexins are also considered to be crucial regulators of monoaminergic neurotransmission. Orexin-induced hyperlocomotion is mediated by the dopaminergic system (Nakamura et al., 2000), the striatal dopaminergic system is altered *in vivo* in human narcolepsy (Eisensehr et al., 2003). The reduced TH mRNA expression was observed in the locus coeruleus of macaques, which was resulted from the age-related decrease of orexin inputs (Downs et al., 2007). These data indicated the regulatory role of orexin-A on dopamine synthesis and TH expression. Here, we demonstrated that orexin-A increased the protein expression of TH accompanied with BDNF both *in vivo* and *in vitro*. Therefore, in addition to indirect modulation by BDNF, the possible direct modulation on TH expression might be another explanation for orexin-A-induced neuroprotection in MPTP-induced parkinsonian mice.

### OX1R Was Involved in Orexin-A Induced Neuroprotection

Orexin-A performed biological effects by OX1R and OX2R with different signaling pathways, such as cAMP-PKA, PLC, PKC, PI3K, and MAPKs (Johansson et al., 2007; Wu et al., 2013; Chen et al., 2015; Kukkonen, 2017). OX1R is 30–100 times more responsive to orexin-A than orexin-B (Marcus et al., 2001). SB334867 is the selective OX1R antagonist, which is at least 50-fold selective over OX2R (Smart et al., 2001). Here, we found that SB334867 could antagonize orexin-A-induced protective effects on MPTP mice (Figures 3–5), which indicated that the protective effect of orexin-A is mainly medicated by OX1R although both of the orexinergic receptors are expressed on dopaminergic neurons of SNpc. Our findings are consistent with other studies showing that orexin-A exerts its function mainly through OX1R. For example, orexin-A increases the firing activity of hippocampal CA1 neurons through OX1R (Chen et al., 2017). Orexin-A signaling via OX1R in the periaquaductal gray area induces analgesia through cannabinoid-induced retrograde inhibition (Esmaeili et al., 2016). Furthermore, orexin-A increases the expression of BDNF through OX1R in the hypothalamus (Harada et al., 2013). Recent study showed that OX1R antagonist completely prevented orexin-A-induced neuroprotective effects against 6-OHDA-induced toxicity in SH-SY5Y cells (Pasban-Aliabadi et al., 2017) and a rat model of PD (Hadadianpour et al., 2017). The signaling pathways related to the effects of orexin-A on BDNF were further investigated. Consistently, we demonstrated that OX1R antagonist SB334867 could also prevent orexin-A-induced upregulation of BDNF (Figure 8A). Furthermore, our data showed that both PI3K inhibitor LY294002 and PKC inhibitor GF109203X abolished the upregulation of BDNF (Figure 8B). These data suggested that the orexin-A-induced upregulation of BDNF is mainly mediated by OX1R via PI3K and PKC signaling pathways. The signaling pathways for BDNF upregulation are in accordance with the neuroprotective pathways of orexin-A in the same cell lines (Pasban-Aliabadi et al., 2017).

## Conclusion

The present study identified that orexin-A exerts neuroprotective effects on MPTP-induced mouse model of PD. The upregulation of BDNF in dopaminergic neurons is involved in the neuroprotective mechanisms of orexin-A. And the effects of orexin-A on BDNF is mediated by OX1R via PI3K and PKC pathways. Our findings provide a rationale for the protection of orexin-A in PD. Further studies are needed to estimate the role of BDNF in orexin-A-induced dopaminergic neuroprotection through BDNF knockdown or knockout selectively in the SNpc.

## Author Contributions

M-FL performed the experiments and wrote the manuscript. M-FL, YX, CL, Y-HL, H-LD, YW, and Y-PP analyzed the data. LC designed the project and revised the manuscript.

## Conflict of Interest Statement

The authors declare that the research was conducted in the absence of any commercial or financial relationships that could be construed as a potential conflict of interest.

## Supplementary Material

FIGURE S1 The original image for the bands of tyrosine hydroxylase (TH) in Figure 7A. The expression of TH in SH-SY5Y cells was detected by immunoblot. The relative molecular weight of TH is about 55 kDa. The central band on the membrane was selected to represent the band of TH in Figure 7A.

FIGURE S2 The original image for the bands of OX1R in Figure 7A. The expression of OX1R in SH-SY5Y cells was detected by immunoblot. The relative molecular weight of OX1R is about 48 kDa.

FIGURE S3 The original image for the bands of pro BDNF and mature BDNF in Figure 7A. The expression of BDNF in SH-SY5Y cells was detected by immunoblot. The relative molecular weight of pro BDNF and mature BDNF are 32 kDa and 14 kDa, separately.

FIGURE S4 The original image for the bands of β-actin in Figure 7A. After the staining of BDNF, the membrane was used for the detection of β-actin. Because of the antigen cross reaction, besides the bands of β-actin, the bands of BDNF were also detected in the membrane.

FIGURE S5 Relative position for the protein bands of OX1R, β-actin, pro BDNF and mature BDNF. The four protein bands are drawn from the same gel. The PVDF membrane was split into two parts for the detection of different proteins. The central bands in the picture were selected to be shown in Figure 7A.

FIGURE S6 The original image for the bands of PI3K in Figure 8B. This membrane includes the protein above 50 kDa. The membrane was used for the detection of PI3K, which has a relative molecular weight of 85 kDa.

FIGURE S7 The original image for the bands of TH in Figure 8B. This membrane with proteins above 50 kDa was also used for the detection of TH. The relative molecular weight of TH is 55 kDa.

FIGURE S8 The original image for the bands of β-actin in Figure 8B. The membrane with proteins between 40 kDa and 50 kDa was used for the detection of β-actin, which serving as a loading control.

FIGURE S9 The original image for the band of BDNF in Figure 8B. The membrane containing proteins less than 40 kDa was used for the detection of BDNF.

FIGURE S10 The original image of Figure 8B. The protein bands of PI3K, TH, BDNF, and β-actin are from one gel. To make the bands concise and potent, we deleted several parallel bands in the center (three bands in orexin-A alone group and one band in orexin-A plus LY294002 group) and two irrelevant bands at the right side. The deleted areas have been pointed out by red frames.

## Abbreviations

BDNF: brain-derived neurotrophic factor
MPTP: 1-methyl-4-phenyl-1, 2, 3, 6- tetrahydropyridine
OX1R: orexinergic receptor 1
OX2R: orexinergic receptor 2
PD: Parkinson’s disease
SNpc: substantia nigra pars compacta
TH: tyrosine hydroxylase
