# Supplementary figures and images for: Orexin-A Exerts Neuroprotective Effects via OX1R in Parkinson’s Disease
Source: Front Neurosci. 2018 Nov 15;12:835. doi: 10.3389/fnins.2018.00835 (PMC6262320; doi:10.3389/fnins.2018.00835)

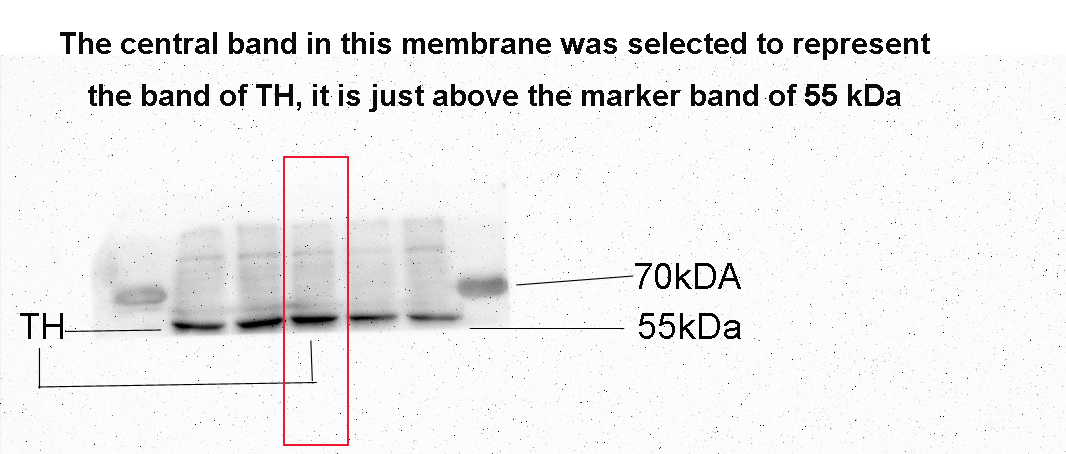

Supplement: FIGURE S1 — The original image for the bands of tyrosine hydroxylase (TH) in Figure 7A. The expression of TH in SH-SY5Y cells was detected by immunoblot. The relative molecular weight of TH is about 55 kDa. The central band on the membrane was selected to represent the band of TH in Figure 7A. [file Image_1.TIF]

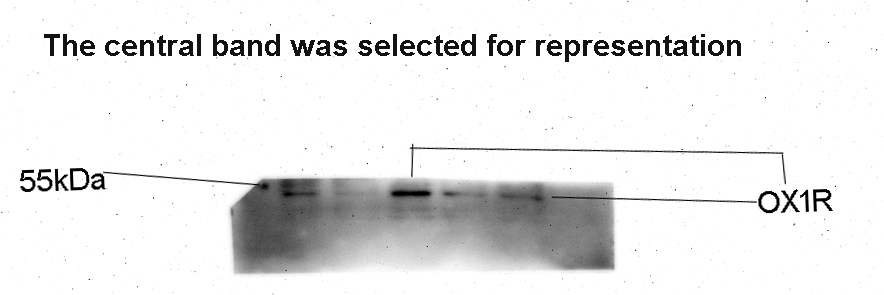

Supplement: FIGURE S2 — The original image for the bands of OX1R in Figure 7A. The expression of OX1R in SH-SY5Y cells was detected by immunoblot. The relative molecular weight of OX1R is about 48 kDa. [file Image_2.TIF]

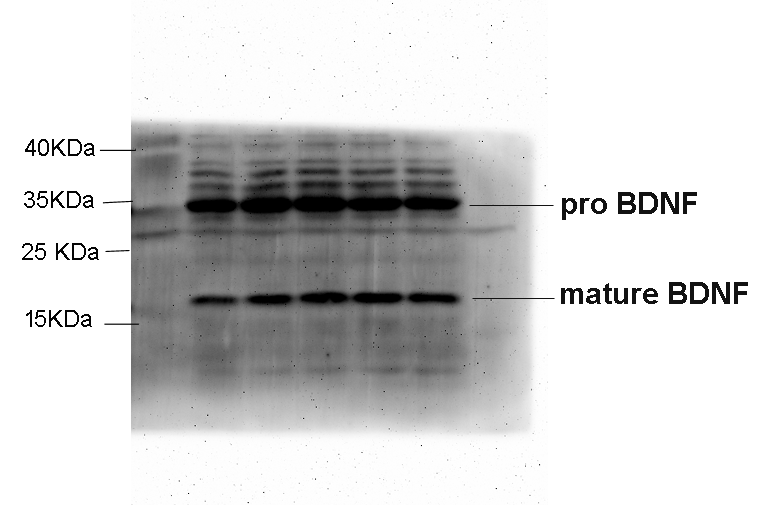

Supplement: FIGURE S3 — The original image for the bands of pro BDNF and mature BDNF in Figure 7A. The expression of BDNF in SH-SY5Y cells was detected by immunoblot. The relative molecular weight of pro BDNF and mature BDNF are 32 kDa and 14 kDa, separately. [file Image_3.TIF]

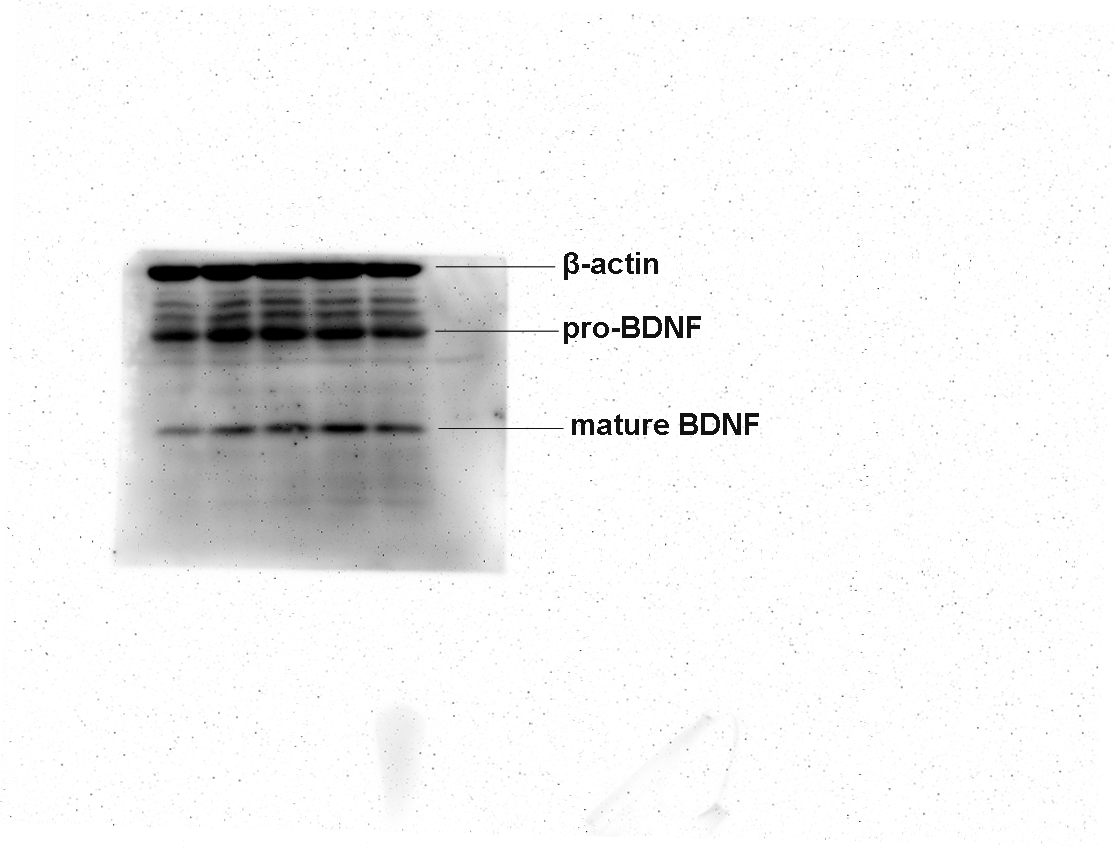

Supplement: FIGURE S4 — The original image for the bands of β-actin in Figure 7A. After the staining of BDNF, the membrane was used for the detection of β-actin. Because of the antigen cross reaction, besides the bands of β-actin, the bands of BDNF were also detected in the membrane. [file Image_4.TIF]

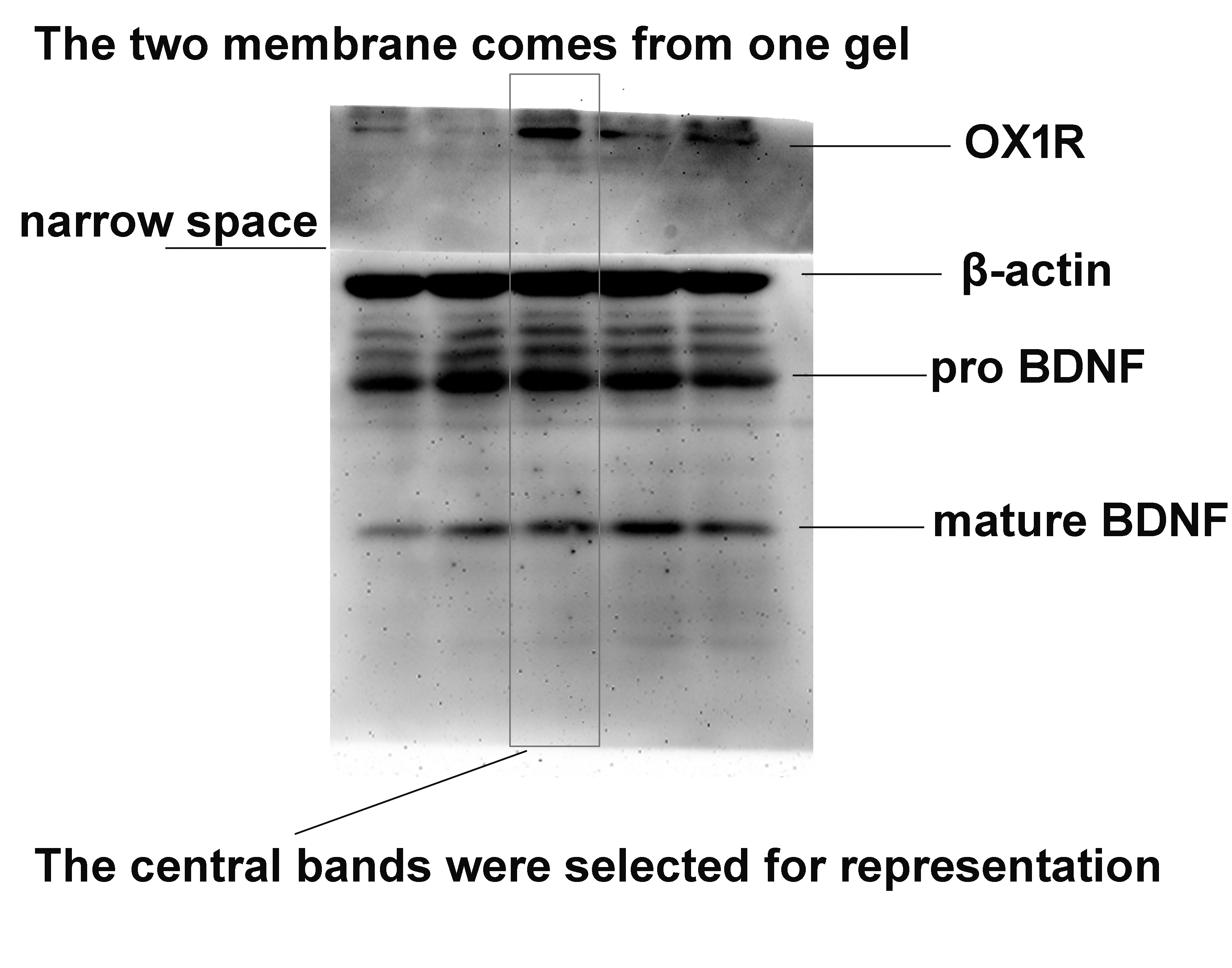

Supplement: FIGURE S5 — Relative position for the protein bands of OX1R, β-actin, pro BDNF and mature BDNF. The four protein bands are drawn from the same gel. The PVDF membrane was split into two parts for the detection of different proteins. The central bands in the picture were selected to be shown in Figure 7A. [file Image_5.TIF]

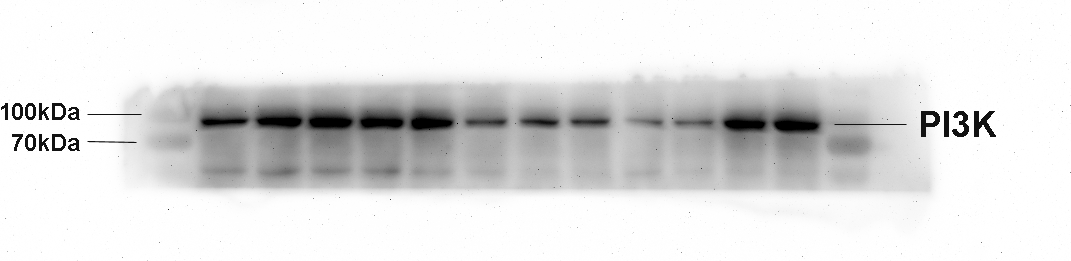

Supplement: FIGURE S6 — The original image for the bands of PI3K in Figure 8B. This membrane includes the protein above 50 kDa. The membrane was used for the detection of PI3K, which has a relative molecular weight of 85 kDa. [file Image_6.TIF]

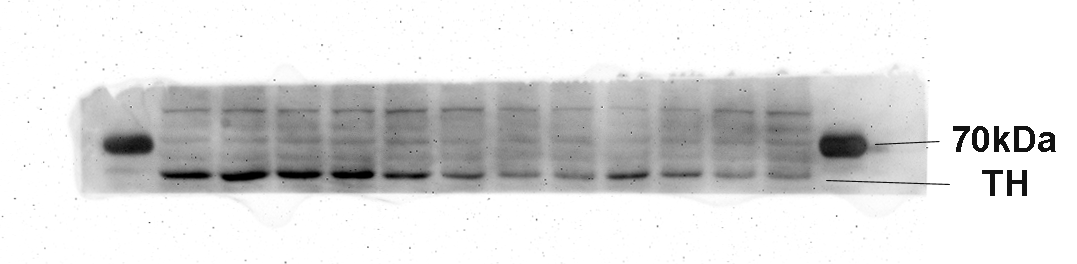

Supplement: FIGURE S7 — The original image for the bands of TH in Figure 8B. This membrane with proteins above 50 kDa was also used for the detection of TH. The relative molecular weight of TH is 55 kDa. [file Image_7.TIF]

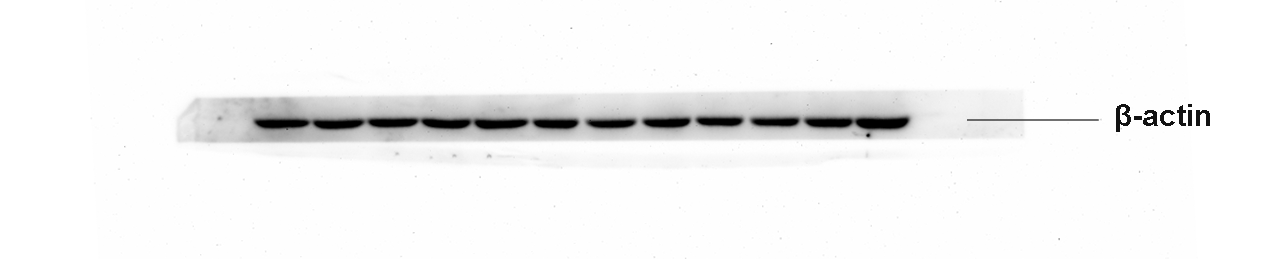

Supplement: FIGURE S8 — The original image for the bands of β-actin in Figure 8B. The membrane with proteins between 40 kDa and 50 kDa was used for the detection of β-actin, which serving as a loading control. [file Image_8.TIF]

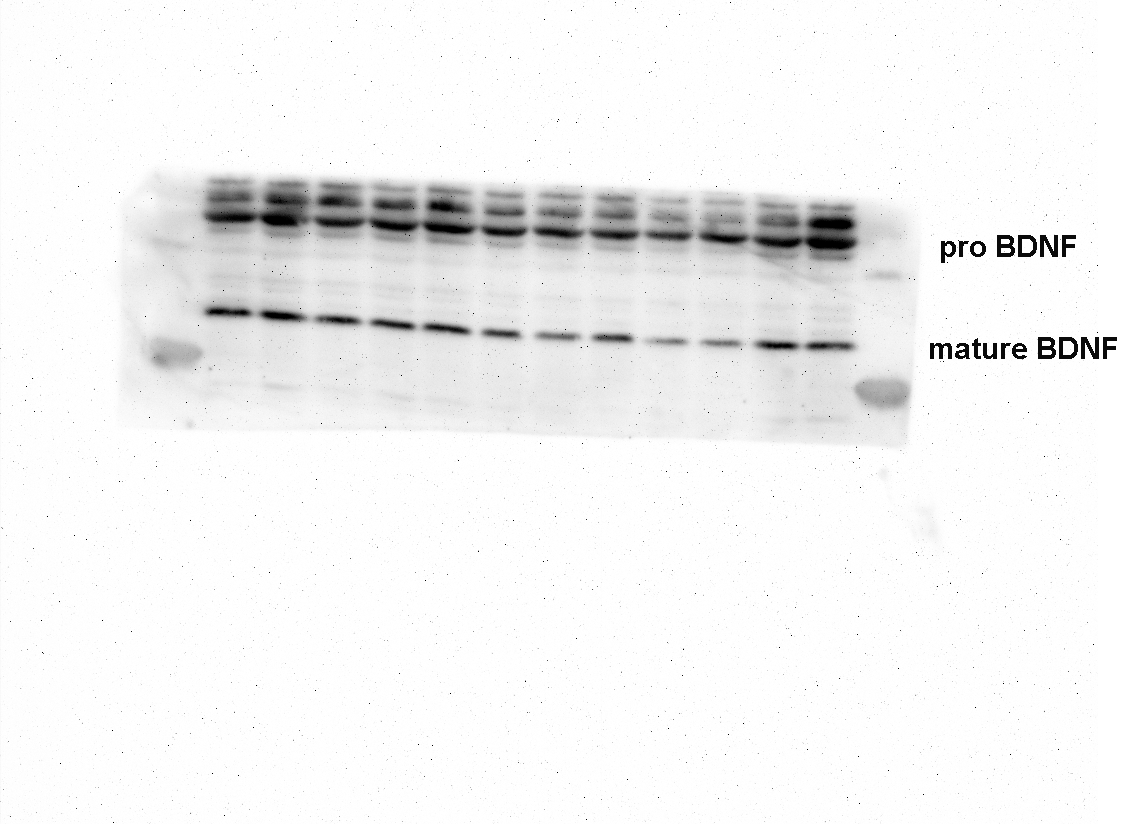

Supplement: FIGURE S9 — The original image for the band of BDNF in Figure 8B. The membrane containing proteins less than 40 kDa was used for the detection of BDNF. [file Image_9.TIF]

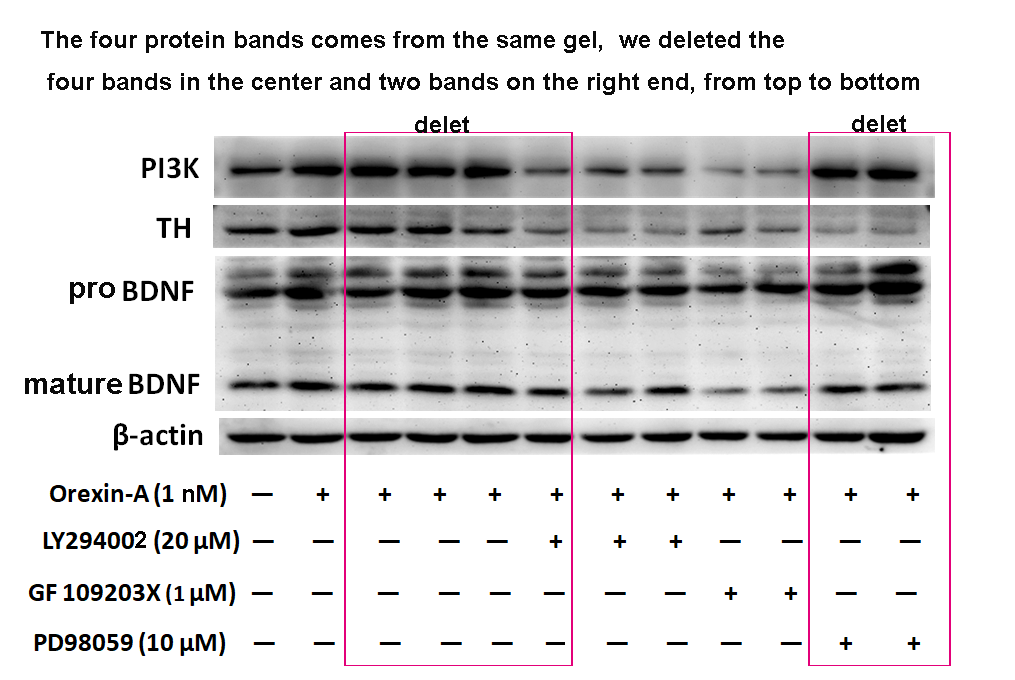

Supplement: FIGURE S10 — The original image of Figure 8B. The protein bands of PI3K, TH, BDNF, and β-actin are from one gel. To make the bands concise and potent, we deleted several parallel bands in the center (three bands in orexin-A alone group and one band in orexin-A plus LY294002 group) and two irrelevant bands at the right side. The deleted areas have been pointed out by red frames. [file Image_10.TIF]
